# Inflammation in Posttraumatic Stress Disorder: Dysregulation or Recalibration?

**DOI:** 10.2174/1570159X21666230807152051

**Published:** 2023-08-07

**Authors:** Kostas Patas, Dewleen G. Baker, George P. Chrousos, Agorastos Agorastos

**Affiliations:** 1 Department of Biopathology and Laboratory Medicine, Eginition University Hospital, Athens, Greece;; 2 Department of Psychiatry, University of California, San Diego (UCSD), La Jolla, CA, USA;; 3 VA Center of Excellence for Stress and Mental Health, VA San Diego Healthcare System, La Jolla, San Diego, CA, USA;; 4 University Research Institute of Maternal and Child Health and Precision Medicine and UNESCO Chair on Adolescent Health Care, National and Kapodistrian University of Athens, Medical School, Aghia Sophia Children's Hospital, Athens, Greece;; 5 Department of Psychiatry, Division of Neurosciences, School of Medicine, Aristotle University of Thessaloniki, Thessaloniki, Central Macedonia, Greece

**Keywords:** Posttraumatic stress disorder (PTSD), immune system, inflammation, neurobiology, microglia, anti-inflammatory agents

## Abstract

Despite ample experimental data indicating a role of inflammatory mediators in the behavioral and neurobiological manifestations elicited by exposure to physical and psychologic stressors, causative associations between systemic low-grade inflammation and central nervous system inflammatory processes in posttraumatic stress disorder (PTSD) patients remain largely conceptual. As in other stress-related disorders, pro-inflammatory activity may play an equivocal role in PTSD pathophysiology, one that renders indiscriminate employment of anti-inflammatory agents of questionable relevance. In fact, as several pieces of preclinical and clinical research convergingly suggest, timely and targeted potentiation rather than inhibition of inflammatory responses may actually be beneficial in patients who are characterized by suppressed microglia function in the face of systemic low-grade inflammation. The deleterious impact of chronic stress-associated inflammation on the systemic level may, thus, need to be held in context with the - often not readily apparent - adaptive payoffs of low-grade inflammation at the tissue level.

## 1 INTRODUCTION

Posttraumatic stress disorder (PTSD) is a chronic and debilitating condition that manifests in the aftermath of one or more profoundly traumatic or life-threatening experiences. Despite the pervasiveness of traumatic events in modern societies worldwide [1], only a substantial minority among the trauma-exposed (ranging from 0.5% to 14.5% across countries) goes on to develop frank PTSD [2]. This suggests that either a disproportionate effect of rather uncommon susceptibility factors or sporadic deficiencies in rather common resilience factors, or more likely a critical mix of both [3-5], might tip the balance on who proceeds to clinical disease.

Rapidly growing evidence indicates that aberrant activity of the immune system/inflammatory reaction may be causally implicated in the pathophysiology of PTSD [6-9], as well as in the association of the disorder with increased risk for somatic manifestations and comorbidities (*i.e*., cardiovascular disease [10] and metabolic syndrome [11]), accelerated biological aging [12] and premature mortality [13]. However, the inference that the immune-inflammatory reaction is dysregulated in stress-related disorders seems to often disregard the critical question of why an inflammatory response is mounted in these disorders in the first place. This question becomes especially pertinent in light of experimental evidence showing that peripheral cytokines can regulate host behavior to optimize the organism’s ability to respond to environmental threats even in the absence of infection [14]. Relatedly, aspects of the immune-inflammatory response, including cell-mediated immunity, are now implicated not only in stress vulnerability but also in stress resilience through both peripheral and central mechanisms of action [15-17]. Therefore, we hereby discuss and re-evaluate the potential role of immune and inflammatory modifications in not merely mediating vulnerability and high medical comorbidity, but also paving the way to adaptation and resilience to PTSD.

## 2 STRESS-ASSOCIATED INFLAMMATION CAN BE BOTH FUNCTIONAL AND DYSFUNCTIONAL

### 2.1 Physical Stress and the Inflammatory Response

The immune system consists of tissues, cell populations, and soluble factors that function to regulate tissue and whole-body homeostasis by means of sensing and responding to physiological and environmental perturbations [18]. A pivotal homeostasis-maintaining process in this context is inflammation – an adaptive response that is initiated by adverse stimuli and dysfunctional systemic, tissue, or cellular states, which are set off either as a result of or in anticipation of the loss of homeostasis [19].

The inflammatory response operates within a dynamic range depending on the nature and abundance of its inducers: while typical inflammation induced by infection and tissue damage lies at one end of the spectrum, graded inflammatory responses of lower magnitude are stretching to the other end [20]. These lower-grade, intermediate inflammatory states that display some but not all of the characteristics of proper inflammation (and are, thus, often termed “parainflammation”) are triggered in response to tissue stress or malfunction and are considered more common but less characterized than the full-fledged inflammatory responses elicited by infection or overt injury [20]. It is thus appreciated that parainflammation and inflammation comprise overlapping components of the continuum of the stress response and extend the adaptive competence of the organism by complementing the homeostatic regulation imparted by the endocrine and autonomic nervous systems [20-23]. How inflammatory signals are functionally embedded in the maintenance of homeostasis at the tissue level is, however, less clear [19].

In a general sense, parainflammation and inflammation are called into action when upstream, tissue-level homeostatic effectors are insufficient or have been overwhelmed in their effort to remove or sequester the source of a disturbance [19, 21]. To this end, a variety of inflammatory mediators, including cytokines, acute phase reactants, and chemokines, are initially produced during innate (*i.e*., non-antigen-specific) immune responses and mobilize other immune cells and responsive tissues. This mobilization is normally proportional to the intensity of the disturbance and lasts for as long as the host needs it to adapt to the new, aberrant conditions and re-establish homeostasis. If these conditions are transient, an equally transient inflammatory response will be actively resolved after it has succeeded in restoring tissue structure and function. Timely inhibition of pro-inflammatory pathways is mediated by intertwined immune, endocrine, and neural regulatory mechanisms that safeguard against undue tissue and cell damage [24, 25]. By contrast, if the aberrant conditions are prolonged (owing to the persistence of the initiating stimuli and/or faulty resolution associated with either an excessive or a subnormal inflammatory response [26]), then a different, chronic type of reaction shifts the organism to different internal set-points. Although the progression from transient to non-resolving inflammatory responses is often not clear-cut [26], the adaptive, physiological aspects of chronic (para)inflammation are gradually and eventually less evident, as opposed to the maladaptive, pathological aspects. It is however presumed that, while often not readily apparent, a physiological counterpart of chronic (para)inflammation is, by and large, present [20]. This may be largely achieved by the fact that inflammatory mediators act on the same cellular functions involved in tissue physiology and homeostasis [19].

### 2.2 Psychological Stress and the Inflammatory Response

Adverse psychological conditions represent no exception with regard to their potential to function as inducers of a wide range of immune responses, including a readily measurable pro-inflammatory state [23]. Indeed, both acute and chronic psychosocial stressors in humans have been reliably associated with low-grade systemic inflammatory responses, largely resembling parainflammation [27, 28]. Although these responses can be unequivocally conceptualized as inherent physical manifestations of vigilance mechanisms towards real, imminent, or perceived threats, sustained low-grade inflammation in response to severe life challenges and enduring psychosocial stressors, such as those described in PTSD and other stress-related disorders ([29-31], see also next section), is rarely interpreted as a dynamic process with inherently pleiotropic - both detrimental and beneficial - implications [23, 32]. Indeed, it is commonly acknowledged that chronic engagement of the endocrine and autonomic stress response systems can affect the homeostatic efficiency of peripheral inflammatory processes towards either chronic disinhibition or excessive inhibition, resulting in systemic inflammation- or immunosuppression-related medical conditions, respectively [33, 34]. This line of work certainly provided valuable insight into the contribution of maladaptive aspects of inflammation to the development of chronic disease, it has however, also bolstered a binary understanding of stress-induced (para)inflammation, wherein mounting of a response is rather uniformly detrimental and its attenuation should be purely beneficial [32, 35].

Similarly, increasing evidence from preclinical models of environmental and psychosocial stress indicates that stress-induced activation of resident microglia in the central nervous system (CNS) leads to mild or modest pro-inflammatory responses that are reminiscent of parainflammation rather than *bona fide*, pathogenic neuroinflammation [36-39]. Cellular and molecular signals associated with stress-induced CNS microdamage [40, 41] are proposed to trigger in this setting a “sterile” microglia response which contributes to a recalibration of behavior in favor of neurorepair, but may nevertheless be prone to premature suppression [42, 43] or low-grade chronicity [38, 41]. On this account, an essentially adaptive CNS response to stressful conditions may be inadequate from the outset or ultimately amount to an unfavorable net effect in some individuals.

## 3 SYSTEMIC INFLAMMATION AND CELLULAR IMMUNOSENESCENCE IN PTSD

### 3.1 Enhanced Innate Immune Responses and Peripheral Inflammation

A growing body of evidence demonstrates a link between PTSD and a heightened systemic inflammatory tone [30, 44]. In particular, meta-analytic research shows that the disorder is cross-sectionally associated with increased levels of the cytokines interleukin-6 (IL-6), IL-1β, tumor necrosis factor-alpha (TNF-α), and interferon-gamma (IFN-γ) [6, 45], as well as of the pro-inflammatory C-C motif chemokine ligands (CCL)3, CCL4 and CCL5 [46]. Consequently, elevation in the levels of the cytokine-responsive acute phase reactant C-reactive protein (CRP) has been observed in individuals suffering from PTSD [6, 47, 48]. Of note, although cytokine mobilization and low-grade acute phase responses are not specific to the diagnosis of PTSD, exposure to psychological trauma has been linked to such pro-inflammatory activity transdiagnostically [49].

Blood-based transcriptomics broadened the scope of immunological understanding in PTSD pathogenesis by showing enhanced expression of numerous genes associated with cytokine/innate immune responses and type I IFN signaling [50], as opposed to weakened expression of genes associated with anti-inflammatory activity [51]. Epigenetic approaches point also to an altered immune function as a result of reduced methylation of immune-related genes in association with increased PTSD symptom severity [52, 53], as well as modifications of leukocyte microRNA expression more permissive to inflammation in PTSD patients [54, 55].

Indirect corroboration of a pro-inflammatory state in the periphery comes also from studies suggesting a blunted immune regulation in PTSD. Regulatory T (Treg) cells are pivotal in establishing an immunological set-point and a decrease in their frequency or function may indicate enhanced effector immune responses. Accordingly, a lower proportion of Treg cells [54, 56] and a less suppressive Treg phenotype in PTSD [57] hint at a disruption of immune homeostasis that may be further facilitating systemic inflammation and possibly pathological autoimmunity. Indeed, patients suffering from PTSD are at an increased risk not only for cardiovascular disease [10, 58] and metabolic syndrome [11], but also for autoimmune disease [59, 60].

Of note, several prospective studies have examined whether inflammation precedes the development of PTSD or *vice versa*. For instance, high pre-trauma CRP [61] or elevated IL-6 immediately after exposure to trauma [62] predict an elevated risk for PTSD. Similarly, over-expression of genes enriched for functions of innate immunity and IFN signaling at baseline has been associated with increased PTSD risk following trauma exposure [50], and a high capacity of leukocytes to produce cytokines upon stimulation has been associated with increases in PTSD symptoms in response to post-trauma severe life events [63]. However, recent studies showed that lower rather than higher levels of TNF-α and IFN-γ in the acute post-traumatic period are associated with subsequent risk for PTSD development [64], partly in a sexually dimorphic manner [65], indicating that, even in prospective designs, disentangling cause and effect may be challenging. In fact, despite the prevalence of plausible pathophysiological links between inflammation and PTSD, current longitudinal research precludes definite conclusions on directionality and causality [66].

### 3.2 Accelerated Aging of Cell-mediated Immunity and the Emerging Positive Role of T Cell Enhancement

A small but growing literature indicates that crucial components of cell-mediated immunity may undergo premature senescence in PTSD patients. It is well-known that functional decline in the T cell and natural killer (NK) cell compartments coexists with parainflammation in aging and aging-related diseases [67-69]. Interestingly, a comparable phenotypic and molecular signature of accelerated immunosenescence is generally evident in stress-related disorders [70, 71], including PTSD [12, 13, 72-75]. Besides neuroendocrine disruption [22], prematurely senescent immune phenotypes could be also partly associated with chronic antigenic stimulation [76, 77], as patients suffering from PTSD, despite being characterized by enhanced innate immune responses, are also at an increased risk for severe infections [78, 79]. In fact, chronic inflammatory signaling, possibly interlinked with oxidative stress, may directly contribute in this setting to T cell and NK cell suppression, similar to what has been described in various non-psychiatric medical conditions [80-82]. Acute phase reactants, particularly CRP, also interfere with antigen presentation, suppress T cell receptor engagement, and inhibit the expansion of antigen-specific T cells [83, 84]. However, it is increasingly apparent that cellular immunosenescence and inflammation represent two sides of the same ambivalent immunological state, one that may engender both functional and dysfunctional modes of adaptation [68, 85].

Beyond their role in host defense, T cells play also a vital role in CNS homeostasis and brain reserve, as shown by a large number of pertinent animal studies, reviewed in [86-88]. As such, it is conceivable that premature T cell senescence in PTSD may deprive the CNS of neuroprotective immune responses during chronic stress and trauma exposure. Indeed, T cell trafficking to the CNS is implicated in homeostatic mechanisms of coping with psychological trauma in preclinical models, and, therefore, targeted augmentation of beneficial CNS-directed T cell responses may constitute a novel approach to enhance resilience to traumatic stress, mainly *via* salutary effects on hippocampal plasticity and neurotrophic balance [89-92]. Importantly, such potentiation of T cell immunity enhances CNS immunosurveillance in stressed animals and attenuates chronic stress-induced behavioral deficits, when procured both before stress exposure (in the form of either active immunization with CNS-derived peptides [89, 90] or by transient depletion of peripheral Treg cells [92, 93]), as well as after exposure to chronic stress (in the form of adoptive transfer of stress-conditioned lymph node cells to chronically stressed lymphopenic animals [91, 94]).

From this perspective, lower Treg proportions and blunted suppressive phenotype in PTSD (see previous section) could be seen as a compensatory moderation of Treg function towards pro-inflammatory T cell effector responses and thereby as an endogenous effort to retain optimal CNS immune surveillance. Indeed, lower Treg percentages are accompanied by higher IFN-γ-producing T helper 1 (Th1) cell percentages in PTSD patients, in part due to a downregulation of microRNAs regulating the expression of the typical Th1 cytokine IFN-γ [54, 95]. Interestingly, elegant preclinical studies demonstrate a central role of systemic IFN-γ signaling, as well as of Th1 polarization of CNS-specific T cells in maintaining immunosurveillance of the CNS in both health and disease [96-98].

Another line of experimental evidence suggests that stress resilience can be achieved through immunization not only with self-antigens but also with non-pathogenic foreign antigens or whole microbes. The environmental saprophyte *Mycobacterium vaccae* is a prime example of a highly immunomodulatory microorganism, which has been repeatedly shown to prevent or ameliorate PTSD-relevant behavioral phenotypes when administered in heat-killed preparations [99-102]. Although it is generally considered that the stress-protective effects of *M. vaccae* are mainly mediated by the induction of Treg cells and an overall anti-inflammatory milieu in animal models, it is likely that the host-microbe interaction leads to a balanced expansion of pro-inflammatory effector T cell populations as well. For instance, human whole blood stimulation with heat-killed *M. vaccae* induced not only an increased release of cytokines (*i.e*., IL-6, TNF-α, as well as the anti-inflammatory cytokine IL-10), but also the upregulation of various adhesion molecules on innate immune cells [103], including the β2 integrins CD11a/CD18 (which are important for monocyte migration [104]) and the costimulatory receptor CD58 (which plays a critical role in T and NK cell activation and proliferation [105]). Importantly, mycobacterial stimulation of human whole blood resulted in increased monocyte expression of pivotal receptors for antigen presentation to CD4^+^ T cells, *i.e*., the major histocompatibility (MHC) class II molecules HLA-DP, HLA-DQ, and HLA-DR, and the T cell costimulatory molecules CD80/ CD86 [103], suggesting that enhanced T cell activation and proliferative capacity is involved in the beneficial immunomodulatory and behavioral effects following immunization with *M. vaccae*.

### 3.3 Multiple Pathways Leading to Peripheral Inflammation in Traumatic Stress

Although numerous biological links between traumatic stress and inflammation have been proposed, the underlying mechanisms are still not completely understood. In general, both genetic components (*e.g*., immunogenetic architecture [106] and sex-specific immune reactivity [107, 108]), as well as environmental/epigenetic influences seem to variably contribute to the inflammatory alterations observed in PTSD patients [7, 9]. Moreover, the timing of the environmental influences (*i.e*., pre- or post-trauma) provides an extra layer of intricacy. For instance, early-life adversities, *i.e*., parental separation, childhood maltreatment, and lower socioeconomic status, play a sizeable role in amplifying immune responses to stressors later in life [109, 110], while at the same time, inflammation can be fostered to some extent by altered lifestyle-related parameters, *i.e*., poor health behaviors, following trauma exposure [111, 112]. As discussed in the following sections, assembling known immunobiological information into converging pathophysiological pathways may not be a straightforward task, especially if certain pathway components concurrently allow for homeostatic adaptation.

### 3.4 Neuroendocrine Perturbations in PTSD

It is widely acknowledged that immune dysfunction can manifest as a consequence of stress-induced disruption of the hypothalamic-pituitary-adrenal (HPA) axis and the autonomic nervous system (ANS) [22, 33]. This is part of a complex, two-way interaction between the central and peripheral limbs of the stress system and the immune system [113-115], which gained particular interest in the pathophysiology of PTSD [116, 117].

On the one hand, post-traumatic ANS imbalance with increased sympathetic and reduced vagal activity [118] may directly augment pro-inflammatory responses [117, 119]. Stress mobilizes pro-inflammatory cytokines in the peripheral circulation [120], in part by activating the transcription factor Nuclear factor kappa-light-chain-enhancer of activated B cells (NF-κB) in mononuclear leukocytes following adrenergic stimulation [121]. Cytokines, however, unfold systemic effects beyond the orchestration of the host immune response and stimulate the secretion of glucocorticoids (GCs), which, although initially reinforce the innate immune system, are subsequently involved in the regulation and, eventually, termination of the inflammatory response [114, 122]. It is, thus, possible that suppressed ability of GCs to regulate inflammation due to low basal GC tone, may lead to untimely disinhibition of cytokine release in PTSD patients, or a subset thereof [123], thereby adding PTSD to a long list of inflammatory states associated with decreased HPA axis activity after major or prolonged stress [124]. Differential GC sensitivity among different immune cell subpopulations and altered GC-mediated transcriptional and post-transcriptional control of immune-related gene expression constitute downstream pathways through which post-traumatic HPA axis dysregulation may facilitate a pro-inflammatory state [116, 117].

At the same time, higher sensitivity of T cell, but not monocyte, proliferation to dexamethasone before military deployment has been associated with increased risk for elevated PTSD symptoms at 6 months post-deployment [125], suggesting that T cell activation deficits associated with increased regulation by GCs pre-exist in some PTSD patients. Lower basal GC signaling after PTSD development in these patients may, thus, in part stimulate T cell activation in a compensatory manner, leading to higher production of T cell-derived cytokines, such as IFN-γ. Indeed, GCs impair the trafficking of stress-protective leukocytes to the CNS, and systemic neutralization of GC signaling both amplified Th1 trafficking into the cerebrospinal fluid (CSF) and mitigated PTSD-like behavioral deficits [92]. In particular, increased GC signaling diminishes Th1 differentiation *via* both T cell-extrinsic and -intrinsic mechanisms [126, 127], suggesting that lower systemic GC tone in PTSD patients may serve Th1 homeostasis and, thus, immune surveillance of the stressed CNS. Nevertheless, under certain conditions, this may also facilitate the manifestation of inflammatory and autoimmune diseases [128].

### 3.5 Immune Genes Associated with PTSD

In addition to endocrine perturbations, genetic variants may also regulate certain aspects of immune reactivity in PTSD. For instance, single-nucleotide polymorphisms within inflammation-related genes (*e.g*., the *CRP* gene) may interact with PTSD to increase systemic inflammation and lead to heightened PTSD symptoms [48, 129]. Interestingly, the genetic association between CRP and PTSD is not unidirectional and appears to be at least in part accounted for by socioeconomic status [130]. Furthermore, genome-wide association data suggest immunogenetic loci, including HLA alleles, to be associated with PTSD [131-133], as well as with intergenerational trauma [134]. Epigenetic variation in the HLA and other genomic regions associated with immunity and inflammation, *i.e*., aryl hydrocarbon receptor repressor, IL-17 signaling, and the complement system, is likewise linked to PTSD risk [135-139]. HLA genes are highly polymorphic and varying combinations of different polymorphisms result in the expression of cell surface proteins with functionally distinct antigen presentation capacities. This leads to an individualized regulation of immune responses that impacts T cell activation, inflammation, as well as susceptibility or resilience to immune-mediated diseases [140]. Therefore, genetic and epigenetic variation in the HLA region may reciprocally shape these immunological domains in PTSD patients as well [106].

Intriguingly, in addition to their conventional role in the immune system, preclinical evidence shows that MHC class I molecules and their receptors are also expressed in the CNS, mediating important functions in neurodevelopment, neuronal plasticity, and stress reactivity [141, 142], suggesting that variation in the HLA class I region in PTSD patients may directly contribute to disease susceptibility or resilience. Similarly, mice lacking MHC class II genes, thus, notably deficient in CD4^+^ T cells, exhibit several behavioral abnormalities, along with suspension of microglia maturation [143]. Of note, microglia constitute the main antigen-presenting cell type in the mature CNS and their level of MHCII expression is linked to the CNS inflammatory response shaping pathology in common neurodegenerative diseases [144]. Certain subsets of MHCII-expressing microglia may nevertheless be able to drive antigen-specific neuroprotection [145, 146], once more indicating that the functional importance and directionality of the observed immunogenetic diversity in PTSD is largely unknown.

## 4 PTSD-ASSOCIATED INFLAMMATION IN THE CNS: TOO MUCH OR TOO LITTLE?

### 4.1 Animal Models and Translational Implications

Sensing of peripheral inflammatory activity by the CNS is believed to be enabled in PTSD by means of several communication pathways leading to perturbations in neurotransmitter metabolism, neural plasticity, cellular reduction-oxidation, microglia-driven neuroinflammation and excitotoxicity [7, 117, 147-150]. These pathways include active transport of peripheral cytokines across the blood-brain barrier (BBB), passive diffusion through leaky regions of the BBB, transmission of peripheral inflammatory signals by virtue of diverse neuroimmune receptors on afferent nerve fibers, and trafficking of mononuclear leukocytes to the CNS in response to chemoattractant signals originating from activated microglia and astrocytes [7, 117, 151].

However, it should be noted that a considerable part of the experimental work describing these pathways is based on the employment of exogenous inflammatory stimuli in otherwise non-stressed animals [152, 153], a model which may adequately recapitulate behavioral and neurobiological aspects of infection or cytokine therapy-induced inflammation in humans, but provides limited construct validity with regard to the pathogenesis of chronic stress-related disease [154]. Importantly, such an exogenous model largely precludes the delineation of potentially preventive or corrective functions of endogenous inflammation in response to the loss of proper neuroendocrine homeostasis elicited by chronic stress [22]. The latter becomes particularly relevant in view of the counterintuitive outcomes – *i.e*., no overall therapeutic effect or even worsening of psychiatric symptomatology – observed in patients with chronic stress-related disorders following anti-cytokine [155-157], nonsteroidal anti-inflammatory drug [158] or low-dose aspirin interventions [159, 160].

A more comprehensive view of PTSD-like behavioral and neurobiological phenotypes may be provided by animal studies employing more ecologically valid environmental stressors [161]. Neuroendocrine responses to such psychological stressors regulate both positively and negatively the production of inflammatory responses within CNS regions, such as the hypothalamus, the hippocampus, and the frontal cortex [162]. In particular, while central catecholamines stimulate the release of IL-1β from microglia through the activation of β2-adrenergic receptors, the stress-induced surge of GCs inhibits the production of CNS cytokines *via* both suppression of noradrenergic locus caeruleus neurons and inhibition of the NF-κB signaling pathway [162]. Animal findings further show that several pro-inflammatory pathways, most prominently the IL-1β signaling pathway, are activated within the CNS in response to both acute and chronic stressors and mediate disruption of hippocampal neurogenesis [163-165], suggesting that PTSD-associated inflammation may act as a negative regulator of neural plasticity and functional connectivity in humans [166]. Of note, the developmental timing of first trauma exposure may add an extra layer of complexity to the relation between neuroinflammation and disease pathogenesis, as data from animal studies show that early life adversity can inhibit homeostatic neuroinflammatory responses during early development, thereby leading to a sensitized immune response and heightened CNS inflammation later in life [167, 168].

Yet, peripherally induced inflammation in adult animals not only reverses stress-induced behavioral changes but also induces dramatic increases in neurogenesis, as well as recovery of hippocampal microglial proliferation following exposure to chronic unpredictable stress [42, 169], indicating a dynamic, context-dependent role of systemic inflammation in stress-related disorders. In fact, the exact effects of short-term *vs*. repeated stress exposure on microglia status, neurogenesis, and associated behavior can be diametrically opposed, that is, an initial period of microglial proliferation and activation may lead to subsequent microglial apoptosis and suppressed neurogenesis [42], with the latter being mediated, at least in part, by enhanced microglial checkpoint expression [170]. This suggests that experimental and therapeutic interventions in this setting should reckon with the CNS inflammatory status [171]. Indeed, aiming at either microglia inhibition or microglia stimulation in preclinical stress models seems to critically depend on whether these cells are in a state of activation or suppression, respectively [42, 43]. In a translational analogy, whereas acute experimental inflammation elicits clinical aspects of depression in healthy subjects [172], it induces mood improvement in severely depressed patients [173]. Moreover, recent preclinical data suggest that the amelioration of chronic stress-induced symptomatology by inflammatory stimulation of hippocampal microglia requires increased synthesis of brain-derived neurotrophic factors through activation of extracellular signal-regulated kinase 1/2 signaling [174].

Interestingly, while prior exposure to a stressor can potentiate microglia pro-inflammatory responses to a subsequent peripheral immune challenge [175], prior exposure to peripherally or intranasally administered innate immune stimuli can prevent rather than precipitate stress-induced behavioral abnormalities *via* microglia stimulation and concomitant increases of TNF-α, IL-6 and IL-1β in the hippocampus and frontal cortex [176-178]. Strikingly, single-event microglia stimulation (pro-inflammatory “vaccination”) in adolescent mice conferred long-lasting protection against heightened neuroinflammatory responses and behavioral abnormalities brought about by chronic stress in adulthood [179]. This prophylactic aspect of sterile inflammatory preconditioning further corroborates the notion that timely and controlled augmentation of inflammation may bear tolerance-inducing clinical benefits in stress-related disorders.

### 4.2 *In vivo* and Postmortem Human Observations

Similarly to peripheral inflammation, microglia activation in humans is not associated with a specific psychiatric diagnosis. Chronic psychosocial stress is instead hypothesized to be a common denominator across diagnoses or patient subgroups [180]. Interestingly, clinical and postmortem human brain data suggest that a PTSD diagnosis can be associated with both amplified and subnormal CNS inflammatory responses (Table **1**). For instance, CSF IL-6 used as a surrogate marker of CNS inflammation showed no consistent alterations or covariation with peripheral IL-6 in PTSD patients [181-183], suggesting that peripheral cytokine signals may not readily or necessarily propagate in the CSF in some patients under unchallenged conditions. By contrast, an immediate and sustained increase of the pro-inflammatory IL-1β, as opposed to a delayed increase of the anti-inflammatory IL-10, in response to a deep pain stimulus was observed in the CSF of combat veterans with PTSD, indicating increased pain-induced neuroinflammatory sensitization [184].

What is further remarkable is that studies exploring parenchymal indications of neuroinflammation identify regionally diminished transcriptional signatures in PTSD, such as decreased *IL1A* gene expression in the dorsolateral prefrontal cortex (dlPFC) [185] and decreased expression of gene sets associated with immune-related pathways and microglia activity in both PFC and amygdala brain regions [186, 187]. Aberrant activity in cortico-amygdala neural circuits has been identified in both PTSD-relevant animal models and individuals with PTSD [188], and recent transcriptomic studies provide compelling evidence of strong microglia enrichments among genes with downregulated expression in these circuits in patients with PTSD relative to neurotypical individuals [187, 189]. Interestingly, co-expression network analyses indicate that while immune-related networks enriched for microglia-specific transcripts are indeed downregulated in the ventromedial PFC, other immune-related networks (*e.g*., enriched for *TNF* and interleukin genes) are largely upregulated in the dlPFC [190], suggesting subregional specificity of neuroimmune suppression in PTSD.

Along the same line, recent data from *in vivo* [^11^C]PBR28 positron emission tomography (PET) brain imaging of the 18-kDa translocator protein (TSPO), a biomarker of microglia activity in humans [191], demonstrate lower rather than higher TSPO availability in prefrontal-limbic regions in PTSD patients [186]. An additional layer of confirmation is provided by a PET study showing significantly higher availability of metabotropic glutamate receptor 5 (mGluR5) in the PFC and ventral striatum of PTSD patients, a finding that was corroborated by upregulation of the expression of SH3 And Multiple Ankyrin Repeat Domains 1 (*SHANK1*), which anchors mGluR5 to the cell surface [192]. Although this study did not report on indices of neuroinflammation, previous work has shown that mGluR5 activation inhibits microglia activation and has a suppressive effect on microglia-associated inflammation [193]. Apart from reduced microglia cell numbers and function, another PET study showed lower [^11^C]SL25.1188 availability in corticolimbic regions in PTSD, suggesting a loss of astrocytes as well [194]. Of note, astrocyte and microglia activation are commonly concurrent in the context of CNS inflammation [195] and astrocytes may be causally linked to PTSD pathogenesis [196].

Strikingly, lower TSPO availability - alluding to compromised microglial function - was associated with both higher blood CRP levels and greater PTSD severity [186]. As long as peripheral CRP contributes to central inflammation in PTSD [197], an inverse association of heightened systemic inflammatory activity with suppressed CNS inflammatory activity is perhaps reminiscent of the premise that chronic (para)inflammation has a physiological counterpart corresponding to tissue stress or malfunction [20, 21]. Given the prevalence of inflammation-targeting approaches in psychiatry and recent failures of both cytokine-specific and blanket anti-inflammatory interventions to separate from placebo in chronic stress-related disorders [156-160], such a re-interpretation may warrant further experimental and prospective investigation.

## 5 RE-INTERPRETATIONS AND CLINICAL IMPLICATIONS

**Fig. (1):**
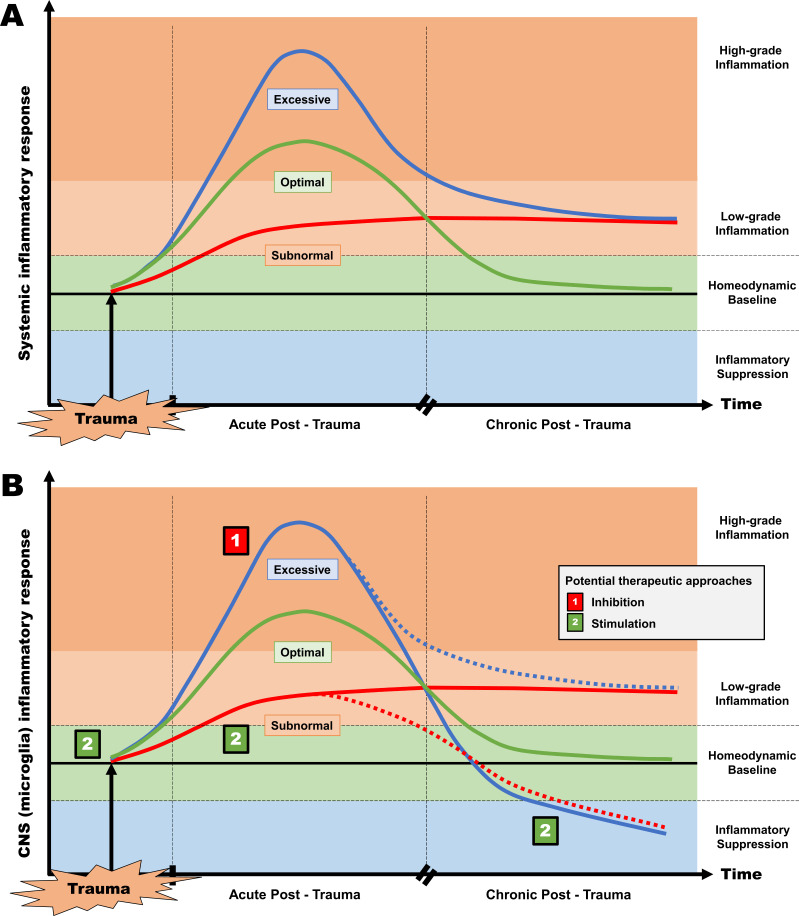
Proposed model represents the dynamic alterations in systemic inflammation and microglia activation status in PTSD based on the type of response in the periphery (**A**) and within the CNS (**B**). Non-resolving, low-grade inflammation in the chronic post-trauma phase may be associated with either an excessive or a subnormal inflammatory response in the acute post-trauma phase in both compartments. However, an excessive or subnormal CNS inflammatory response in the acute phase may also pave the way to a premature suppression of microglia activity. Accordingly, both anti-inflammatory approaches (acutely and subacutely post-trauma) and pro-inflammatory approaches (in the form of either pre-trauma immunization or post-trauma boosters) may be therapeutically pertinent, depending on whether the microglia compartment is in a state of overactivation or suppression, respectively.

The findings reviewed above reveal that the distinction of adaptive *vs*. dysregulated inflammatory responses in PTSD – and possibly other stress-related disorders – is not always readily drawn. According to converging evidence from several experimental and observational studies, it may be argued that some PTSD patients are characterized by systemic parainflammation in the face of microglia-associated neuroinflammatory suppression. Preclinical and clinical data further suggest that both anti-inflammatory and pro-inflammatory approaches may be therapeutically pertinent, depending on the contextual dynamics of stress-associated inflammation within the CNS rather than the incremental shift to a higher inflammatory set-point in the periphery (Fig. **1**).

Maintenance of bodily tissue integrity and function depends on graded inflammatory and physiological self-reactive responses which are contingent upon the degree of tissue stress or malfunction that is being experienced [20, 198, 199]. This seems to extend to immune activation that is evoked by perturbations of CNS homeostasis [86, 200, 201]. What largely differentiates advantageous from disadvantageous immune activity is the intensity, timing, and context of its elicitation. In fact, neuroimmune responses to psychological stress may be better conceptualized on the basis of parainflammation (*i.e*., low-grade inflammatory responses to homeostatic threats) rather than on the basis of established concepts of neuroinflammation-driven pathology, as seen in CNS disease, injury or infection [39]. On this account, not every detectable aspect of immune and inflammatory alteration is expected to be of pathophysiological or therapeutic relevance to PTSD.

Notably, inflammation has neuroprotective properties widely described in the general field of neuroimmunology [37, 202, 203]. Elevations in circulating and topical cytokines, microglia activation, and recruitment of peripheral leukocytes into areas of the CNS are not necessarily undesirable [36]. In this regard, the magnitude of neural tissue malfunction can determine the magnitude of reciprocal immune-to-neural communication, whereby timely bouts of inflammation and controlled recruitment of systemic immune cells to the CNS may actually pave the way for neuro repair processes [204, 205]. A notable example of how peripheral and central inflammation may exert neuroprotection is the increased production and release of neurotrophic factors by mononuclear leukocytes [206, 207] and/resident microglia [174], a feature of immune activation with potential therapeutic relevance to CNS disorders [203, 208]. Fig. (**2**) summarizes possible mechanisms of immune stimulation leading to enhanced stress resilience.

Along with this notion, a dual role of low-grade IL-6 and CRP elevations in stress-related disorders is being gradually recognized [32, 209, 210]. This is conceptually important as both biomarkers have been extensively employed in many disease contexts to infer an underlying state of pathogenic inflammation rather than homeostasis-restoring inflammation. Of note, IL-6 is a prototypical cytokine with demonstrated functional pleiotropy and context-dependent pro- as well as anti-inflammatory properties [211-213], which, among others, connects peripheral regulatory processes with the CNS [214, 215]. Perhaps unsurprisingly, PTSD symptom severity has been associated with both higher [45] and lower levels [216, 217] of peripheral IL-6. A global blockade of IL-6 signaling would thus conceivably be expected to have heterogeneous, *i.e*. both on- and off-target effects, especially if patients are not stratified to have higher or lower levels of the target biomarker.

Indeed, a recent clinical study in patients undergoing allogeneic hematopoietic stem cell transplantation showed that global blockade of IL-6 receptors (*i.e*., both membrane-bound and soluble) by the monoclonal antibody tocilizumab was associated with worsening of depressive symptomatology [157], presumably due to indiscriminate blockade of IL-6 signaling in the periphery, increased BBB permeability of tocilizumab under conditions of significant peripheral inflammation and unrestricted activity of unbound IL-6 across the BBB [157, 218]. A similar note of caution is raised by recent safety signals about a potential link spontaneous depression and suicidal behavior with the use of specific monoclonal antibodies inhibiting the IL-17 receptor [219] or lymphocyte migration across the BBB [220], suggesting that abrupt reductions in systemic inflammatory signaling or CNS-directed immune reactivity may trigger adverse psychiatric effects in susceptible individuals [221].

Of direct relevance to the timing and efficacy of immune interventions in PTSD patients, recent prospective data suggest that an inflammatory response immediately after trauma exposure (as assessed by levels of TNF-α and IFN-γ) may mediate resilience rather than susceptibility to the disorder [64]. In view of the often opposing actions of both studied cytokines within the CNS [222, 223], the results of this study are setting the stage for further research on potentially adaptive aspects of acute peritraumatic inflammation. Indeed, follow-up work indicates that lower peritraumatic pro-inflammatory activity is prospectively associated with increased risk for nonremitting PTSD in women, while higher peritraumatic pro-inflammatory activity may confer PTSD resilience in men [65]. Preliminary prospective evidence likewise suggests that the pro-inflammatory C-X3-C motif chemokine ligand 1 (CX3CL1) is a PTSD resilience marker in US military service members [224]. Congruently, mice lacking CX3CL1 display cognitive dysfunction as a result of impaired synaptic plasticity and neurogenesis, while treatment with soluble CX3CL1 is able to, at least in part, restore these deficits [225]. If corroborated by further prospective and back-translational studies, a “paradigm shift” could be therefore reinforced whereby an initially deficient rather than excessive inflammatory response may drive in some cases the development of chronic PTSD [226]. Such a scenario could be seen as less counterintuitive in light of immunological studies showing that non-resolving inflammation may emanate from an inflammatory response that begins subnormally [26], along with preclinical evidence of proactive exposure to sterile inflammatory stimuli conferring tolerance to stress-induced behavioral and neurobiological abnormalities [176-179].

Critically, however, specific targeting of key components of non-resolving signaling pathways that converge to maintain systemic parainflammation in PTSD will eventually be needed in the chronic post-trauma phase. As genetic factors and inflexible environmental demands can progressively antagonize or undermine the adaptive capacity of an inflammatory response, endogenous inflammatory stimulation directed to the CNS can be gradually rendered suboptimal and inevitably maladaptive, especially for somatic tissues [113, 115, 227]. It is thus expected that nuanced anti-inflammatory and other immune-modulating interventions will be needed to curtail the increased medical burden associated with PTSD [228-230].

## CONCLUSION

Initiation of inflammatory responses by disruptions of cellular and tissue homeostasis is deeply embedded in our biology, likely owing to the evolutionary prominence of such disruptions as forerunners of potential infection [231-233]. By extension, adverse psychosocial conditions, especially enduring or repeating ones leading to profound disruptions of neuroendocrine and immune cell homeostasis, could be seen as distinct environmental inducers of “preemptive” inflammation with both destructive (*i.e*., defense of the host) and restorative (*i.e*., defense of tissue homeostasis) capacities. Indeed, we now know that many inflammatory mediators can also operate as expanded homeostatic signals when a regulated variable deviates beyond the homeostatic range [19]. This may be an often overlooked and thus underexplored physiological aspect of parainflammation in stress-related disorders, one that argues for a balanced appreciation of both the value and the cost of the inflammatory response – and, as such, for a pragmatic and more personalized therapeutic targeting of the process.

## Figures and Tables

**Fig. (2):**
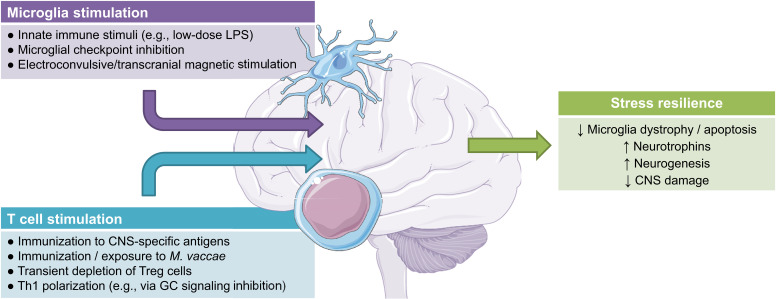
Potential therapeutic and/or prophylactic immunostimulatory approaches leading to enhanced stress resilience. References are provided in the text. **Abbreviations:** LPS: lipopolysaccharide; CNS: central nervous system; Treg: regulatory T cells; GC: glucocorticoid.

**Table 1:** CSF and CNS parenchymal inflammatory indices in PTSD.

**Study Type**	**References**	**Sample Size**	**Methods**	**Highlights**	**Limitations**
*In vivo* (serial CSF and plasma)	Baker *et al.*, 2001 [181]	PTSD: 11 Controls: 8	ELISA	• ↑ CSF IL-6 in PTSD • ↔ plasma IL-6 • Negative correlation between CSF and plasma IL-6 in PTSD	• Sample size • Only males
*In vivo* (CSF) after paroxetine treatment	Bonne *et al.*, 2011 [182]	PTSD: 16 Controls: 11	ELISA	• ↔ CSF IL-6 pretreatment • ↔ CSF IL-6 posttreatment	• Sample size • No peripheral measurements
*In vivo* (diurnal CSF and plasma)	Agorastos *et al.*, 2019 [183]	PTSD: 12 Combat controls: 12 Non-combat: 11	ELISA	• ↔ diurnal CSF IL-6 • ↔ diurnal plasma IL-6 • Circadian blunting of plasma IL-6 in all combat-exposed participants	• Sample size • Only males
*In vivo* (CSF) after deep pain stimulus	Lerman *et al.*, 2016 [184]	PTSD: 10 Controls: 11	ECL	• ↑ CSF IL-1β post-injection in PTSD • Delayed ↑ CSF IL-10 post-injection in PTSD	• Sample size • Only males • No peripheral measurements
Postmortem brain gene expression	Morrison *et al.*, 2019 [185]	PTSD: 12 MDD: 25 Controls: 13	RT-qPCR	• ↓ dlPFC *IL1A* in PTSD • ↔ dlPFC *IL1A* compared to MDD	• Most PTSD cases comorbid with MDD • No peripheral measurements
*In vivo* (PET)	Bhatt *et al.*, 2020 [186]	PTSD: 23 Controls: 26	Brain PET and immuno-turbidimetry	• ↓ Prefrontal-limbic TSPO availability in PTSD • Negative correlation between TSPO availability and plasma CRP in PTSD	• Single time-point peripheral measurements • PTSD sample with low medical burden due to exclusion criteria for PET
Postmortem brain gene expression	Bhatt *et al.*, 2020 [186]	PTSD: 22 Controls: 22	RT-qPCR	• ↓ PFC *TSPO* in females with PTSD • ↓ PFC *TNFRSF14* and *TSPOAP1* in females with PTSD	• Inherent differences in clinical characteristics between postmortem and PET samples
Postmortem brain transcriptomics	Jaffe *et al.*, 2022 [187]	PTSD: 107 MDD: 109 Controls: 109	RNA-Seq	• ↓ PFC and amygdala immune-related pathways in PTSD • ↓ PFC and amygdala microglia in PTSD	• Potential under-representation of female donors
Postmortem brain transcriptomics	Girgenti *et al.*, 2021 [189]	PTSD: 52 MDD: 45 Controls: 46	RNA-Seq	• ↓ dACC microglia in PTSD • ↓ OFC and female sgPFC *UBA7* in PTSD	• Microglia quantified by gene expression deconvolution
Postmortem brain transcriptomics	Logue *et al.*, 2021 [190]	PTSD: 38 MDD: 32 Controls: 24	RNA-Seq	• ↓ vmPFC microglia-related network in PTSD • ↑ dlPFC immune-related network in PTSD	• PTSD cases comorbid with MDD were also included
*In vivo* (PET)	Gill *et al.*, 2022 [194]	PTSD: 13 Controls: 17	Brain PET	• ↓ mPFC and ventral striatum astrocytes in PTSD	• Sample size • Possibly confounded by medication
*In vivo* (PET)	Deri *et al.*, 2021 [232]	World Trade Center responders: 20	Brain PET	• Positive correlation between PFC-hippocampal TSPO availability and PTSD symptom severity	• Subsyndromal PTSD • Lack of control group
*In vivo* (PET)	Toczek *et al.*, 2019 [233]	PTSD: 9 Controls: 7	Systemic and brain PET	• ↔ FDG signal in the aorta, spleen, bone marrow, or amygdala • Positive correlation between the amygdala, bone marrow, and splenic FDG signal in all participants	• Sample size • Possible selection bias (young subjects)

**Abbreviations:** ↑ increased, ↔ not different, ↓ decreased compared to a healthy control group (unless otherwise specified); CSF: cerebrospinal fluid; PFC: prefrontal cortex; dlPFC: dorsolateral prefrontal cortex; dACC: dorsal anterior cingulate cortex; OFC: orbitofrontal cortex; sgPFC: subgenual prefrontal cortex; vmPFC: ventromedial prefrontal cortex; PET: positron emission tomography; TSPO: 18-kDa translocator protein; TNFRSF14: TNF Superfamily Member 14; TSPOAP1: TSPO Associated Protein 1; UBA7: Ubiquitin-Like Modifier-Activating Enzyme 7; FDG: [^18^F]fluorodeoxyglucose; ELISA: enzyme-linked immunosorbent assay; ECL: electrochemiluminescence; MDD: major depressive disorder; RT-qPCR: reverse transcription quantitative real-time PCR; RNA-Seq: RNA sequencing.

## LIST OF ABBREVIATIONS

ANS: Autonomic Nervous System
CNS: Central Nervous System
CRP: C-reactive Protein
CSF: Cerebrospinal Fluid
GCs: Glucocorticoids
HPA: Hypothalamic-pituitary-adrenal
IFN-γ: Interferon-gamma
IL-6: Interleukin-6
NK: Natural killer
PET: Positron Emission Tomography
PTSD: Posttraumatic Stress Disorder
TNF-α: Tumor Necrosis Factor-alpha
